# Blood Pressure Variability and Outcomes Across Antihypertensive Regimens

**DOI:** 10.1161/HYPERTENSIONAHA.126.27140

**Published:** 2026-06-25

**Authors:** Yue Qiao, Zihan Sun, Eric L. Harshfield, Xunming Ji, Hugh S. Markus, Wenbo Zhao

**Affiliations:** Department of Neurology, Xuanwu Hospital, Capital Medical University, Beijing, China (Y.Q., W.Z.).; Stroke Research Group, Department of Clinical Neurosciences, University of Cambridge, United Kingdom (Z.S., E.L.H., H.S.M., W.Z.).; Beijing Institute for Brain Disorders, Capital Medical University, Beijing, China (X.J.).

**Keywords:** blood pressure, calcium channel blockers, cardiovascular diseases, diuretics, propensity score

## Abstract

**BACKGROUND:**

Blood pressure (BP) variability (BPV) is associated with cardiovascular risk beyond mean BP. Whether first-line antihypertensive regimens differentially affect BPV and cardiovascular outcomes remains uncertain.

**METHODS:**

We conducted a target trial emulation using pooled individual participant data from 2 randomized trials: ACCORD-BP (Action to Control Cardiovascular Risk in Diabetes Blood Pressure) and SPRINT (Systolic Blood Pressure Intervention Trial). Propensity score matching was used for pairwise comparisons of RAS (renin–angiotensin system) inhibitors, calcium channel blockers (CCBs), and diuretics, as monotherapy or in combination. Follow-up was initiated at a predefined baseline medication assessment. The primary outcome was visit-to-visit systolic BPV, assessed using variation independent of the mean. Secondary outcomes included major adverse cardiovascular events and its individual components.

**RESULTS:**

The final cohort included 5779 participants (median of 12 BP measurements and median follow-up of 3.5 years), among whom 2754 were included in the matched analysis. CCB-based regimens were consistently associated with significantly lower systolic BPV compared with both RAS inhibitor-based and diuretic-based regimens. This association was consistent across monotherapy (CCB versus RAS: β=−1.341 [95% CI, −1.930 to −0.752]; *P*<0.001) and combination therapies (CCB+diuretic versus RAS+diuretic: β=−1.299 [95% CI, −1.852 to −0.747]; *P*<0.001). In contrast, RAS inhibitor–based and diuretic-based regimens demonstrated comparable BPV profiles. A total of 215 (3.7%) major adverse cardiovascular events were observed in the overall cohort. No significant differences in major adverse cardiovascular event or individual components were observed across comparisons.

**CONCLUSIONS:**

Among high-risk hypertensive patients receiving target-driven BP management, CCB-based regimens were associated with lower visit-to-visit systolic BPV compared with RAS inhibitor–based and diuretic-based regimens. However, these differences were not accompanied by detectable differences in cardiovascular outcomes.

**Novelty and Relevance**

**What Is New?**

This is the first study to apply a target trial emulation framework with propensity score matching to compare visit-to-visit systolic blood pressure variability (BPV) across first-line antihypertensive drug classes using pooled individual participant data from SPRINT (Systolic Blood Pressure Intervention Trial) and ACCORD-BP (Action to Control Cardiovascular Risk in Diabetes Blood Pressure).
Calcium channel blocker-based regimens were consistently associated with lower visit-to-visit systolic BPV than both RAS (renin-angiotensin system) inhibitor–based and diuretic-based regimens under comparable achieved mean BP.

**What Is Relevant?**

Although calcium channel blocker-based regimens were associated with lower visit-to-visit systolic BPV, no statistically significant differences in major adverse cardiovascular events were observed across antihypertensive regimens.
These findings have direct implications for antihypertensive drug selection guidelines in patients with high-risk hypertension.

**Clinical/Pathophysiological Implications**

Calcium channel blocker-based regimens, despite being associated with significantly lower visit-to-visit BPV, were not associated with significantly reduced major adverse cardiovascular events risk compared with RAS inhibitor–based or diuretic-based regimens, indicating that regimen-specific BPV differences alone may not be sufficient to inform antihypertensive drug selection.

Hypertension is the most prevalent modifiable risk factor for cardiovascular disease and remains a leading contributor to global morbidity and mortality.^1,2^ Robust evidence from randomized trials has demonstrated that antihypertensive therapy markedly reduces the risk of major adverse cardiovascular events (MACE), and current guidelines prioritize achieving recommended mean blood pressure (BP) targets as the principal therapeutic objective.^3,4^ However, even among individuals who achieve these stringent targets, a substantial residual cardiovascular risk persists.

BP variability (BPV), reflecting fluctuations in BP over time, has emerged as a risk marker for stroke, myocardial infarction, and mortality beyond mean BP, with multiple meta-analyses have demonstrated consistent associations with adverse cardiovascular outcomes irrespective of average BP control.^5,6^ Although current guidelines endorse multiple first-line antihypertensive classes, such as ACE (angiotensin-converting enzyme) inhibitors/angiotensin receptor blockers, CCBs (calcium channel blockers), thiazide-like diuretics, these agents are widely considered interchangeable with respect to mean BP reduction and clinical utility.^3,4,7^ However, pharmacological differences among these agents may result in distinct effects on BPV, leading to meaningful heterogeneity in BPV despite similar mean BP control.^8^ Whether specific antihypertensive regimens confer incremental vascular protection by minimizing BPV, particularly within the context of target-driven BP management, remains an important but unresolved clinical question.

Previous comparisons between different antihypertensive regimens have been hampered by methodological limitations. Randomized controlled trials, while providing high-level evidence, often employ standardized protocols that fail to account for individualized drug selection reflective of clinical practice, thereby limiting the ability to evaluate regimen-specific differences in BPV and clinical outcomes.^8^ Conversely, observational studies, while more reflective of real-world prescribing, are susceptible to confounding by indication and other biases that complicate causal inference.^9^ As a result, the comparative effects of commonly used antihypertensive strategies on BPV and cardiovascular outcomes have not been systematically evaluated within a unified analytic framework designed to mitigate confounding under contemporary BP treatment paradigms.^10^

To address these gaps, we conducted a pooled individual participant-level analysis of the SPRINT (Systolic Blood Pressure Intervention Trial) and the ACCORD-BP trial (Action to Control Cardiovascular Risk in Diabetes Blood Pressure). By emulating randomized comparisons of antihypertensive treatment strategies within these trials and applying propensity score–based methods under an intention-to-treat (ITT) framework, we primarily aimed to compare visit-to-visit BPV across antihypertensive regimens under comparable mean BP control, and secondarily to evaluate cardiovascular outcomes associated with these regimens.

## Methods

### Data Availability

The data used in this analysis are available from the National Heart, Lung, and Blood Institute BioLINCC repository (https://biolincc.nhlbi.nih.gov) on application. The analytic code is available from the corresponding author on reasonable request.

### Ethics Statement

The SPRINT and ACCORD-BP trials received institutional review board approval at all participating centers, and all participants provided written informed consent before enrollment. The present secondary analysis of de-identified, publicly available data was approved by the Cambridge Psychology Research Ethics Committee (reference PRE.2025.052).

### Study Design

This study was a patient-level pooled analysis of data from the SPRINT and ACCORD-BP. The designs and primary results of both trials have been reported previously.^11,12^ SPRINT enrolled 9361 participants aged ≥50 years with systolic BP (SBP) between 130 and 180 mm Hg and elevated cardiovascular risk, defined by the presence of clinical or subclinical cardiovascular disease, chronic kidney disease, a Framingham 10-year cardiovascular risk ≥15%, or age of 75 years or older; patients with diabetes or prior stroke were excluded. ACCORD-BP enrolled 4733 participants with type 2 diabetes, aged 40 to 79 years, who demonstrated high cardiovascular risk due to evidence of cardiovascular disease, significant atherosclerosis, albuminuria, left ventricular hypertrophy, or at least 2 additional risk factors, such as dyslipidemia or smoking. In both trials, participants were randomized to intensive (target SBP <120 mm Hg) versus standard (target SBP <140 mm Hg) BP treatment strategies.

Although β-blockers were used by a substantial proportion of participants in both trials, they were frequently prescribed for coexisting cardiovascular conditions, such as coronary artery disease or heart failure, rather than for primary management of hypertension.^7,13^ To minimize confounding by indication and to focus on treatment strategies relevant to first-line BP control, the present analysis concentrated on 3 guideline-recommended antihypertensive classes: RAS (renin-angiotensin system) inhibitors (ACE inhibitors and angiotensin receptor blockers), CCBs, and diuretics (thiazide, loop, and potassium-sparing diuretics).

The data sets for this analysis were obtained from the National Heart, Lung, and Blood Institute’s Biologic Specimen and Data Repository Information Coordinating Center (BioLINCC, biolincc.nhlbi.nih.gov). Both trials received institutional review board approval at all participating centers, and all participants provided written informed consent. Additional ethics approval was not required for this secondary analysis of deidentified data.

### Target Trial Emulation and Analytic Framework

This study was conducted within a target trial emulation framework to compare alternative antihypertensive treatment strategies using individual patient-level data from SPRINT and ACCORD-BP trials. The target trial was conceptualized as a hypothetical randomized trial assigning participants with treated hypertension to specific antihypertensive drug classes or combinations and following them for BPV and MACE.

In both trials, antihypertensive regimens were frequently adjusted during the initial 3 months after randomization to achieve target SBP levels. To minimize the influence of treatment titration on BPV estimation, and taking into account the availability of antihypertensive medication data in each trial, we defined the baseline treatment (time zero) as the medication regimen at the 3-month visit in SPRINT and the 12-month visit in ACCORD-BP.

Treatment strategies were therefore defined based on stabilized medication regimens after the initial titration period, with follow-up initiated at the baseline medication assessment. The primary analysis corresponded to an ITT estimand, while additional per-protocol (PP) analyses were performed to assess the robustness of the findings.

### Participants

Participants were drawn from SPRINT and ACCORD-BP. The present analysis included participants who were alive and free of MACE at a predefined baseline treatment assessment, defined as the 3-month visit in SPRINT and the 12-month visit in ACCORD-BP. Participants were excluded if they met any of the following criteria: (1) use of β-blockers at the predefined baseline treatment assessment; (2) occurrence of a major cardiovascular event before the predefined baseline; (3) fewer than 3 BP measurements between predefined baseline and the occurrence of MACE or the end of follow-up; or (4) absence of any antihypertensive therapy at the predefined baseline treatment assessment.

### BP Measurements and Variability Calculation

In both trials, BP was measured at each clinic visit using validated automated devices (Omron 907XL or Omron 907). In SPRINT, automated office BP measurements were obtained under both attended and unattended conditions across study sites, whereas ACCORD adopted only the former. Participants were seated and 3 consecutive readings were obtained with their average recorded as the visit BP.^12^ Visit-to-visit BPV was calculated using measurements obtained from the baseline medication assessment time point (3 months for SPRINT and 12 months for ACCORD) until a major cardiovascular event occurred or until trial completion.^14^ In SPRINT, following the initial titration phase, scheduled clinic BP assessments were conducted at ≈3-month intervals. In ACCORD-BP, visit frequency transitioned to 4 monthly intervals after the first year of follow-up. At both respective time zero points, BP levels were broadly stable and aligned with the intended targets in the intensive and standard treatment arms, consistent with the completion of initial treatment titration. In this study, mean SBP was calculated as the arithmetic mean of all SBP values during the same period.

Four metrics of BPV were calculated: (1) SD of visit-to-visit BP; (2) coefficient of variation (CV), calculated as SD divided by mean BP; (3) average real variability (ARV), defined as the mean absolute difference between consecutive BP measurements; and (4) variation independent of mean (VIM), calculated as SD divided by mean BP raised to a power k, with k estimated from the population-specific regression of log (SD) on log (mean BP). VIM was prespecified as the primary metric to ensure the assessed variability was independent of mean BP levels.^15^

### Outcome Ascertainment

The primary analytic outcome of this study was visit-to-visit systolic BPV, assessed using the VIM, which was prespecified as the principal BPV metric given its relative independence from mean BP. Additional BPV metrics, including the SD, CV, and ARV, were examined in sensitivity analyses to assess the robustness of the findings.

Secondary outcomes included the time to first occurrence of MACE, defined as a composite of cardiovascular death, nonfatal myocardial infarction, and nonfatal stroke, as well as each individual component of the composite outcome. Time-to-event was calculated from the predefined baseline medication assessment to the first occurrence of a component event, death from any cause, or end of follow-up, whichever occurred first. In both SPRINT and ACCORD-BP, cardiovascular outcomes were systematically ascertained at scheduled study visits and adjudicated using standardized, centrally reviewed protocols based on prespecified clinical criteria.^14,16^

### Statistical Analyses

Continuous variables are presented as mean±SD or median (interquartile range [IQR]), and categorical variables as counts and percentages. Baseline characteristics were compared between groups using independent *t* tests or Mann-Whitney *U* tests for continuous variables and χ^2^ tests for categorical variables, as appropriate.

Propensity score matching (PSM) was used to balance baseline characteristics between medication regimen groups. PSM was performed using nearest-neighbor matching with a 1:1 ratio without replacement. For each pairwise medication regimen comparison, propensity scores were estimated using multivariable logistic regression models with the medication regimen as the dependent variable and baseline covariates listed in Tables S1 and S2 as explanatory variables. A caliper width of 0.1 standard deviations of the logit of the propensity score was applied to ensure high-quality matches. After matching within each trial, the matched cohorts from SPRINT and ACCORD-BP were pooled for subsequent analyses. The quality of matching was assessed by examining standardized mean differences for all covariates in the matched sample, with successful balance defined as standardized mean difference <0.1 for all variables. Propensity score distributions between treatment groups were visualized using density plots.

The primary analyses followed an ITT principle, analyzing participants according to their baseline medication assignment regardless of subsequent medication changes. Linear regression models were employed to compare systolic BPV metrics (SD, CV, ARV, and VIM) and mean SBP during follow-up between antihypertensive regimens, with results reported as β coefficients with 95% CIs. We used Cox proportional hazards regression models to estimate hazard ratios (HR) and 95% CIs for the association between medication regimens and cardiovascular outcomes. The proportional hazards assumption was assessed through visual inspection of log-log survival plots and Schoenfeld residuals. Event rates were calculated as the number of events per 100 person-years of follow-up.

Sensitivity analyses were conducted using a PP approach. In the PP analysis, participants who switched or discontinued their baseline antihypertensive regimen during follow-up were censored at the time of medication change. To assess the consistency of findings across trials that differed systematically in diabetes status, additional ITT sensitivity analyses were performed separately within SPRINT and ACCORD-BP for all pairwise regimen comparisons. Formal trial-by-treatment interaction testing was also performed in the pooled data set. The PP analysis was performed using the same propensity score-matched cohorts as the ITT analysis. To account for residual covariate imbalance after PSM, additional covariate-adjusted Cox proportional hazards models and linear regression models were conducted to assess the robustness of the findings.

All statistical analyses were performed using R (version 4.5.1) software. Given the multiple pairwise comparisons, Bonferroni correction was applied with statistical significance defined as a 2-sided *P*<0.008 (0.05/6) for primary analyses and *P*<0.05 for secondary analyses.

## Results

### Study Population and Baseline Characteristics

**Figure 1.**
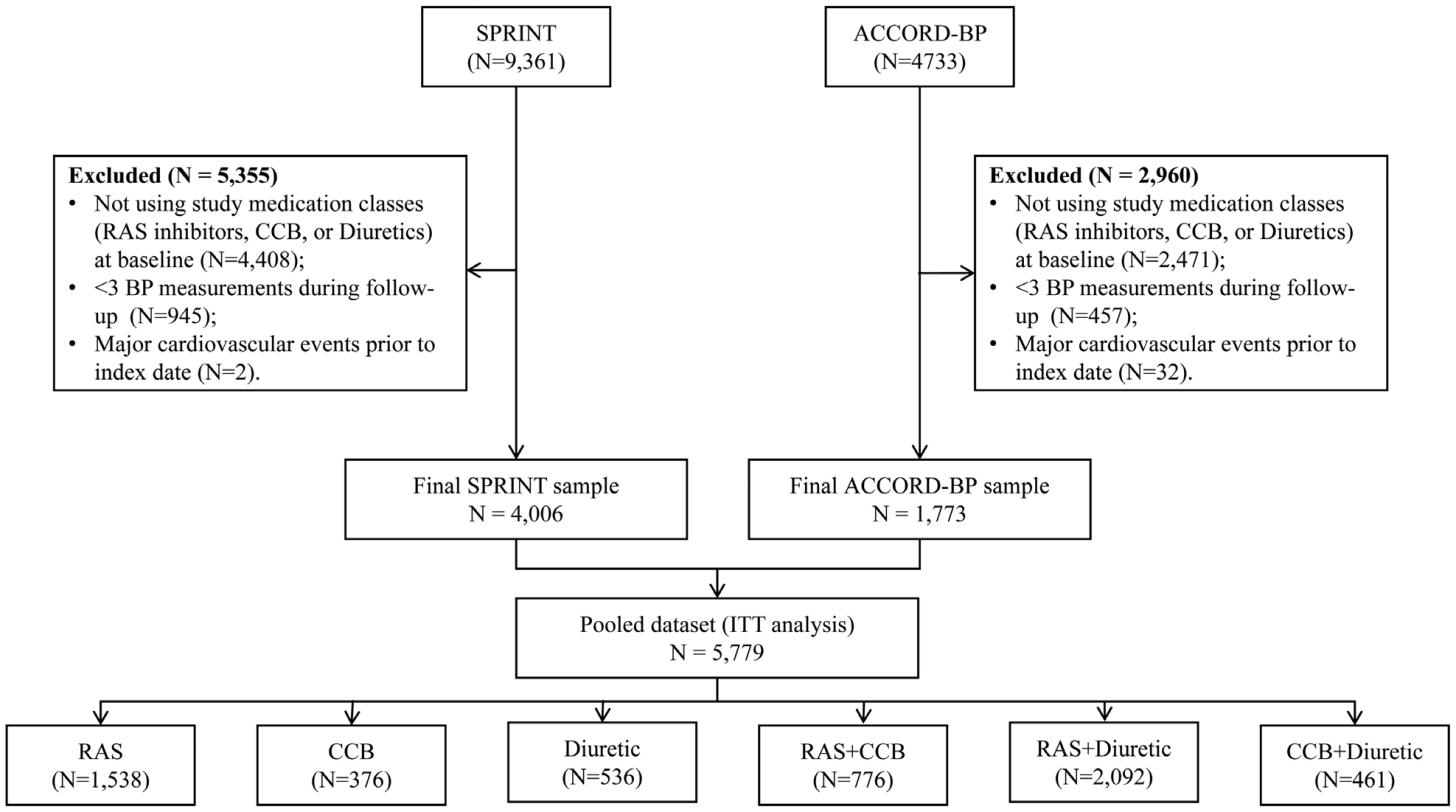
**Study design and participant flow.** Study flow diagram showing participant selection from SPRINT (Systolic Blood Pressure Intervention Trial; N=9361) and ACCORD-BP (Action to Control Cardiovascular Risk in Diabetes Blood Pressure; N=4733), resulting in a final pooled cohort of 5779 participants allocated to 6 antihypertensive regimen groups for the intention-to-treat analysis. BP indicates blood pressure; CCB, calcium channel blockers; ITT, intention-to-treat; and RAS, renin-angiotensin system.

Of the 14 094 participants enrolled in SPRINT (n=9361) and ACCORD-BP (n=4733), 5779 were ultimately included in the present analysis (4006 from SPRINT and 1773 from ACCORD-BP; Figure 1). The primary reason for exclusion in both trials was the absence of qualifying antihypertensive regimens composed of RAS inhibitors, CCBs, or diuretics at baseline (SPRINT: n=4408; ACCORD-BP: n=2471), followed by insufficient BP measurements during follow-up (SPRINT: n=945; ACCORD-BP: n=457) and a prior major cardiovascular event (SPRINT: n=2; ACCORD-BP: n=32). Baseline characteristics of the unmatched cohort are summarized in Table 1 and Table S3. Significant between-trial differences were observed across most variables, largely driven by the by-design difference in ACCORD-BP and SPRINT, though both populations represented high-risk hypertensive cohorts with substantial cardiovascular burden. The mean age of the study population was 65.9±8.9 years, 39.7% were women, and 57.4% were non-Hispanic White. Among monotherapy users, RAS inhibitors were most common (26.6%), followed by diuretics (9.3%) and calcium channel blockers (6.5%). For combination therapy, RAS inhibitors plus diuretics represented the largest group (36.2%), followed by RAS inhibitors plus CCB (13.4%) and CCB plus diuretics (8.0%). Mean SBP during the follow-up was 128.0±10.6 mm Hg overall and similar between ACCORD-BP (126.9±11.2 mm Hg) and SPRINT (128.5±10.3 mm Hg). The median number of BP measurements available per participant for BPV calculation was 12.0 (IQR, 10.0–14.0) overall, with 11.0 (IQR, 10.0–14.0) in SPRINT and 14.0 (IQR, 13.0–15.0) in ACCORD-BP.

**Table 1.**
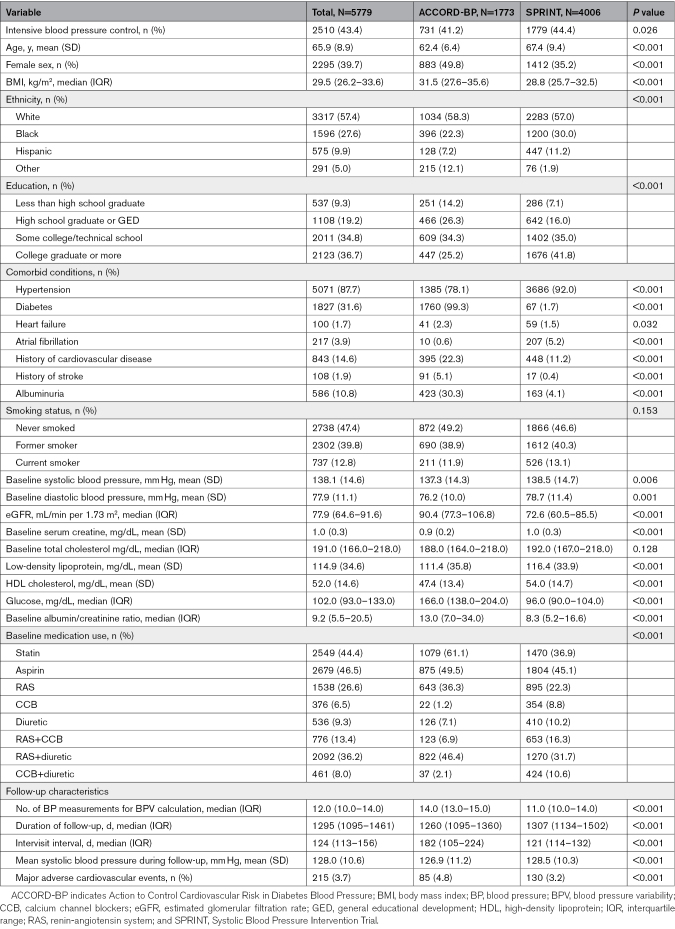
Baseline Characteristics of Participants from SPRINT and ACCORD-BP Included in the Analysis

Substantial differences in baseline characteristics were observed across medication groups before matching (Table S3). Participants receiving combination therapy had higher baseline SBP (139.4–141.3 mm Hg) than those receiving monotherapy (135.7–136.6 mm Hg). The prevalence of diabetes ranged from 6.9% among CCB monotherapy users to 42.7% among RAS inhibitor users (*P*<0.001), while a history of cardiovascular disease ranged from 10.2% to 22.3% across regimens (*P*=0.001). Atrial fibrillation was more prevalent among participants receiving CCB-based regimens (6.2%) compared with other regimens (2.8%–4.6%; *P*<0.001). In contrast, history of stroke and heart failure did not differ significantly between groups. Mean SBP during follow-up was broadly comparable across antihypertensive regimens. Minor differences of mean SBP were observed in selected combination therapy comparisons; however, the overall effects were small and did not indicate systematic differences in achieved BP control between treatment strategies (Table S4).

After 1:1 PSM without replacement, the matched cohort consisted of 2754 participants. Baseline characteristics of the matched cohort are summarized in Tables S1 and S2. Adequate covariate balance was achieved across treatment groups, with standardized mean differences below 0.1 for the vast majority of baseline characteristics. Baseline SBP was well balanced across treatment groups, with no clinically meaningful differences observed. Propensity score distributions demonstrated substantial overlap between groups, supporting the validity of the matching procedure (Figure S1).

### BPV Across Antihypertensive Regimens

In the primary ITT analysis of the matched cohort, antihypertensive regimens differed significantly with respect to visit-to-visit systolic BPV (Table 2; Table S5). CCB monotherapy was consistently associated with lower BPV than RAS inhibitor monotherapy, including lower SBP-VIM (β=−1.341 [95% CI, −1.930 to −0.752]; *P*<0.001), with concordant findings across SBP-SD, SBP-CV, and SBP-ARV. Similarly, CCB monotherapy demonstrated significantly lower BPV compared with diuretic monotherapy across multiple metrics, including SBP-VIM (β=−0.882 [95% CI, −1.438 to −0.327]; *P*=0.002), SBP-SD, and SBP-CV, despite similar mean SBP levels. In contrast, BPV did not differ between diuretics and RAS inhibitors across all 4 BPV metrics or mean SBP.

**Table 2.**
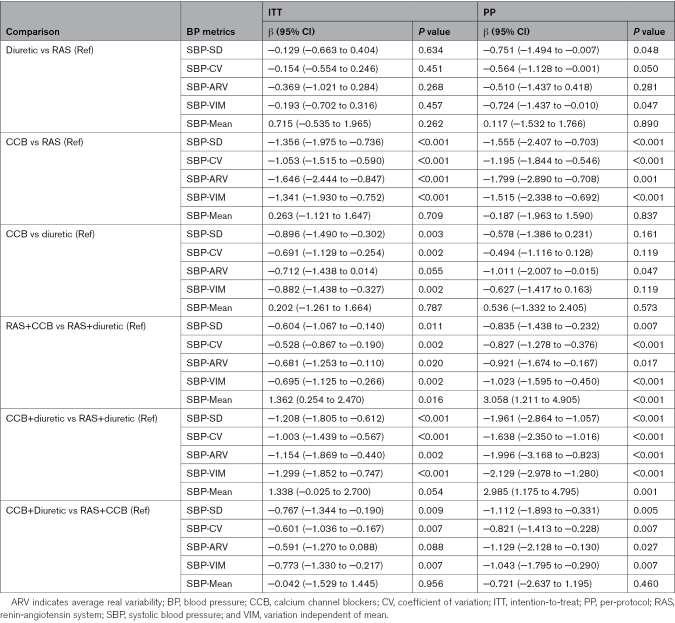
Associations of Antihypertensive Regimens With Visit-to-Visit Systolic Blood Pressure Variability

Among combination regimens, RAS inhibitor plus CCB therapy was associated with lower BPV than RAS inhibitor plus diuretic therapy, particularly for SBP-VIM (β=−0.695 [95% CI, −1.125 to −0.266]; *P*=0.002; Table 2). The largest BPV differences were observed when comparing CCB plus diuretic regimen with RAS inhibitor plus diuretic therapy, with significantly lower SBP-VIM (β=−1.299 [95% CI, −1.852 to −0.747]; *P*<0.001) and consistent reductions across other BPV metrics. In addition, CCB plus diuretic regimen was associated with lower BPV than RAS inhibitor plus CCB regimen, although this difference was attenuated when assessed by SBP-ARV. In trial-specific ITT analyses, the BPV-lowering advantage of CCB-based regimens was predominantly observed in SPRINT but attenuated and nonsignificant in ACCORD-BP (Table S6). However, formal interaction testing did not show statistically significant heterogeneity between SPRINT and ACCORD-BP for the principal BPV comparisons.

PP sensitivity analyses yielded results consistent with the primary ITT analysis (Table 2). In the PP analysis, the association between CCB-based regimens and lower BPV was maintained or even strengthened compared with the ITT analysis, with CCB monotherapy showing consistently lower BPV than both RAS inhibitors and diuretics, and similar patterns observed for combination regimens.

### Major Adverse Cardiovascular Events

**Figure 2.**
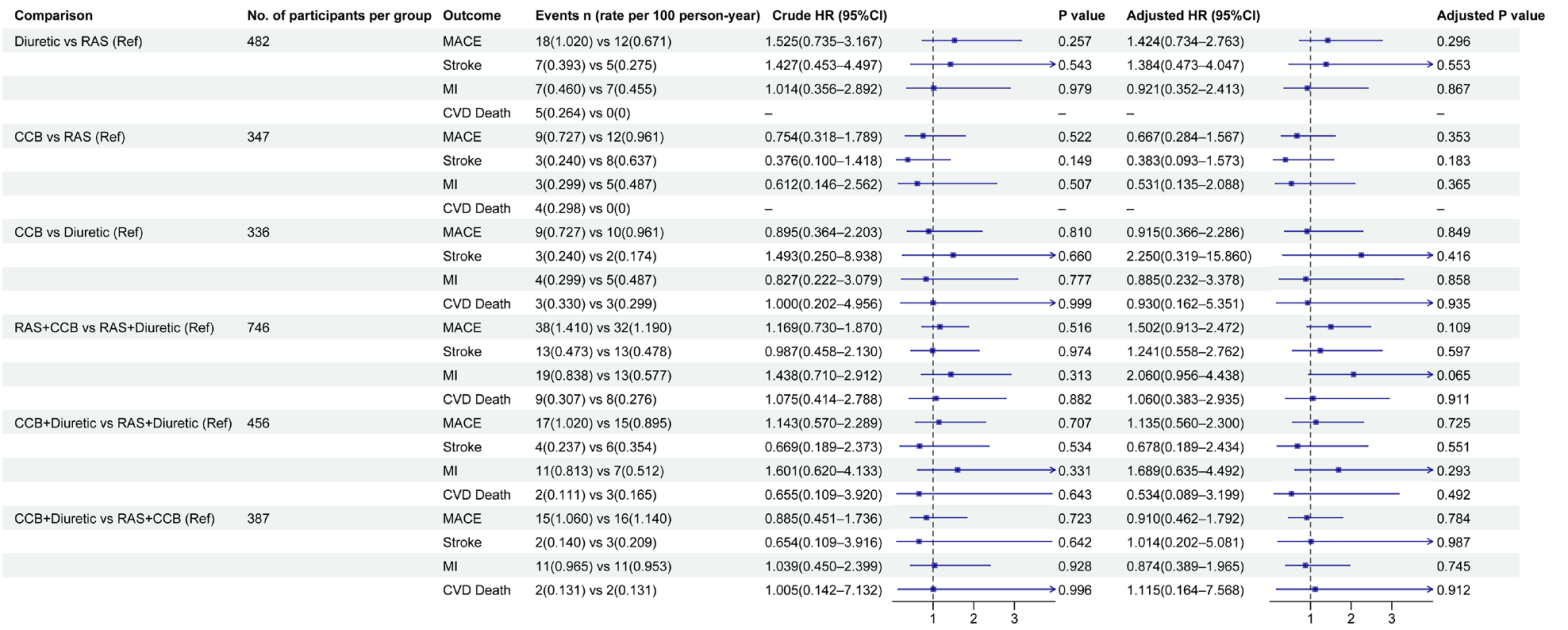
**Antihypertensive regimens and major adverse cardiovascular events (MACE) in the intention-to-treat (ITT) analysis.** Forest plot of crude and covariate-adjusted hazard ratios (HRs; 95% CI) for MACE and its individual components across 6 pairwise antihypertensive regimen comparisons in the propensity score-matched ITT cohort. Events are presented as n (rate per 100 person year). Adjusted models included age, sex, education, ethnicity, treatment allocation, baseline systolic blood pressure, estimated glomerular filtration rate, history of cardiovascular disease, heart failure, statin use, and aspirin use. CCB indicates calcium channel blockers; CVD Death, cardiovascular death; HR, hazard ratio; MI, myocardial infarction; and RAS, renin-angiotensin system.

Over a median follow-up of 3.5 years (IQR, 3.0–4.0 years), a total of 215 (3.7%) MACE events occurred in the overall cohort. In the matched ITT cohort, no statistically significant differences were observed in the risk of MACE across any pairwise medication comparisons in either crude or covariate-adjusted Cox proportional hazards models (Figure 2; Figure S2). Among monotherapy users, the risk of MACE was comparable between participants treated with RAS inhibitors and those receiving diuretics (HR, 1.53 [95% CI, 0.74–3.17]; *P*=0.257) or CCB (HR, 0.75 [95% CI, 0.32–1.79]; *P*=0.522). When comparing CCB with diuretic monotherapy, no significant difference in MACE risk was observed (HR, 0.90 [95% CI, 0.36–2.20]; *P*=0.810). Comparable findings were observed for combination therapies, with no significant differences between RAS inhibitor plus diuretic and RAS inhibitor plus CCB regimens, or between CCB plus diuretic and other combination regimens (each *P*>0.05), trial-specific analyses of MACE similarly showed no significant regimen-specific differences in either SPRINT or ACCORD-BP (Table S7).

Analyses of individual outcome components, including nonfatal myocardial infarction, nonfatal stroke, and cardiovascular death, similarly showed no significant associations with specific antihypertensive regimens. PP analyses yielded consistent results, with no significant differences in MACE or its individual components across medication regimens in either crude or covariate-adjusted models (Figure S3).

## Discussion

In this patient-level pooled analysis of SPRINT and ACCORD-BP, leveraging standardized BP measurements, adjudicated outcomes, and a target trial emulation framework with PSM, we found that CCB-based regimens were associated with significantly lower systolic BPV compared with both RAS inhibitor-based and diuretic-based regimens, whether used as monotherapy or in combination. In contrast, diuretic-based and RAS inhibitor-based regimens exhibited comparable BPV profiles. However, despite clear differences in BPV under comparable mean BP control, no corresponding differences in MACEs were observed. These findings demonstrate that, in this patient population, differences in pharmacological modulation of BP stability were not accompanied by differences in cardiovascular outcomes.

Although the associations between antihypertensive regimens, BPV, and MACEs have been widely investigated, prior studies have largely relied on observational designs and have therefore remained vulnerable to confounding by achieved BP levels and treatment selection. As a result, it has been uncertain whether regimen-specific differences in BPV reflect intrinsic pharmacological properties of antihypertensive therapies or merely residual confounding related to BP control. The present study advances the field by reframing BPV as a treatment-related phenotype rather than solely a prognostic marker. By leveraging the randomized trial infrastructure of SPRINT and ACCORD-BP and applying a target trial emulation framework with PSM, this study is able to compare BPV across antihypertensive regimens under target-driven BP management with comparable achieved mean BP. This approach allowed us to compare differences in BP stability that extend beyond mean BP reduction. Importantly, despite consistent and clinically meaningful differences in BPV across regimens, these differences were not accompanied by corresponding differences in MACE. This dissociation suggests that regimen-related differences in BPV do not necessarily translate into incremental cardiovascular benefit once mean BP is adequately controlled.

Our findings align with previous evidence demonstrating that CCBs significantly reduce visit-to-visit systolic BPV compared with other first-line antihypertensive classes, including both RAS inhibitors and diuretics.^17,18^ The observation that CCB monotherapy reduced BPV compared with both RAS inhibitors and diuretics is consistent with the unique pharmacological properties of CCBs, which is characterized by sustained vasodilation and may attenuate short-term BP fluctuations more effectively than other drug classes.^10^ In contrast to previous meta-analyses, this study found no significant BPV differences between diuretics and RAS inhibitors.^15^ This discrepancy may reflect several factors. First, the SPRINT and ACCORD-BP cohorts comprised high-risk populations, among whom the vascular remodeling and endothelial protective effects of RAS inhibitors may attenuate BPV under protocol-driven BP control, potentially diminishing differences observed in lower-risk cohorts from prior meta-analyses.^19,20^ Second, by applying rigorous PSM to balance baseline characteristics across treatment groups, this analysis may provide a less confounded estimation of regimen-specific effects than prior studies, which were often confounded by differential patient phenotypes, nonrandomized treatment allocation, or unadjusted observational data. Nonetheless, important clinical and methodological differences inherent to the pooling of 2 distinct trial populations cannot be fully eliminated. In addition, differences in BPV metrics, assessment intervals, and analytical models could also contribute to divergent results.^10^ Trial-specific analyses further indicated that CCB-based regimens with lower BPV was more evident in SPRINT and attenuated in ACCORD-BP. This difference may reflect reduced precision after within-trial matching, differences in baseline treatment patterns, and possible effect modification related to diabetes status, where hyperglycemia-driven arterial stiffening and autonomic neuropathy impair the baroreflex-mediated buffering through which CCBs stabilize BPV.^10,21^

Despite the differential effects on systolic BPV, our study found no significant differences in MACE across antihypertensive regimens. These findings are partially consistent with posthoc analyses from SPRINT, in which visit-to-visit BPV (assessed from 3 to 12 months) showed no association with cardiovascular events when mean BP was adequately controlled; however, the same study found that the highest BPV quintile was associated with nearly doubled all-cause mortality risk (adjusted HR, 1.92 [95% CI, 1.22–3.03]).^22^ Two additional SPRINT analyses reached divergent conclusions: Cheng et al^23^ demonstrated that visit-to-visit BPV independently predicted all-cause mortality after accounting for Framingham risk score, while Mezue et al^24^ showed that diastolic BPV independently predicted cardiovascular outcomes and hypoperfusion-related adverse events. That 3 analyses of the same data set reached different conclusions underscores the methodological sensitivity of findings in this field, including metric selection, outcome definition, and follow-up window.^25^ However, this contrasts with prior meta-analyses suggesting that amlodipine-based regimens were associated with both lower BPV and lower risk of stroke and cardiovascular events.^18,26^ Moreover, Mendelian randomization evidence, leveraging genetic variants to minimize confounding, have consistently shown that genetically proxied CCBs use is associated with reduced cardiovascular risk, including lower incidence of stroke and cardiovascular mortality; similar evidence for other antihypertensive classes (eg, RAS inhibitors, β-blockers) reveals heterogeneous protective effects.^27^ Of note, the present study focuses specifically on visit-to-visit BPV measured at clinic visits; other dimensions of BPV, including day-by-day home BP variability and within-day variability, represent distinct phenotypes that may carry independent prognostic significance and warrant separate investigation. Several factors may contribute to this discrepancy. The largest benefits in previous studies were observed when CCBs were compared against β-blockers,^28^ whereas RAS inhibitors served as the primary comparator in this study. Moreover, both SPRINT and ACCORD-BP employed intensive SBP targets (<120 mm Hg), which may have diminished the prognostic value of BPV.^29^ Additionally, our analysis was further constrained by low event rates (eg, overall stroke incidence 1.5%) and relatively short follow-up (median, 3.5 years), consistent with the ASCOT study suggesting that BPV-mediated cardiovascular benefits may require longer time horizons to become manifest.^15,30^ Notably, no cardiovascular deaths occurred in the RAS inhibitor arm after matching, underscoring the limited statistical power to detect regimen-specific differences in mortality.

Whether BPV directly contributes to MACE or merely reflects underlying vascular pathology remains debated. Prior studies have shown that although CCBs effectively attenuate BPV over time, these drug-induced reductions do not fully mitigate the cardiovascular risk associated with baseline BPV.^31,32^ Similarly, evidence from the J-HOP study indicates that BPV correlates with target organ damage independent of mean BP, supporting its role as a marker rather than a direct mediator of vascular injury.^33^ Consistent with these findings, our results confirm that CCB-based regimens are more effective than RAS inhibitors in stabilizing systolic BPV; however, the lack of associated differences in cardiovascular events suggests that BPV should be interpreted cautiously, particularly in the context of rigorous mean BP control.

### Perspectives

This study demonstrates that among high-risk hypertensive patients managed within intensive, target-driven BP treatment paradigms, CCB-based antihypertensive regimens produce systematically lower visit-to-visit systolic BPV compared with RAS inhibitor–based and diuretic-based regimens. However, these pharmacologically driven differences in BP stability did not translate into statistically detectable reductions in MACE under contemporary BP control standards. These findings suggest that, in the context of intensive target-driven BP management, regimen-specific differences in BPV may not independently confer incremental cardiovascular protection over the time horizons examined, whether such benefits emerge with longer follow-up remains to be established. From a clinical standpoint, these results argue against selecting antihypertensive regimens based solely on their BPV-lowering properties in high-risk patients with well-controlled mean BP. Meanwhile, efforts to develop antihypertensive regimens that simultaneously optimize mean BP control and minimize BPV, and to evaluate their long-term cardiovascular consequences across diverse populations, remain warranted to advance more precise and individualized hypertension management.

### Limitations

This study has several limitations. First, although we leveraged a target trial emulation framework, this analysis remains a posthoc comparison within trials originally designed to evaluate SBP targets rather than antihypertensive drug classes. Accordingly, regimen-specific associations cannot be considered equivalent to those derived from randomized drug-class comparisons. Second, while PSM achieved good balance across measured covariates, residual confounding from unmeasured factors, such as medication adherence, dose titration intensity, BP measurement context, and differing baseline assessment timepoints (3 months in SPRINT versus 12 months in ACCORD-BP), cannot be entirely excluded. Third, pooling ACCORD-BP and SPRINT introduced systematic between-trial heterogeneity in diabetes status that could not be fully addressed by within-trial matching. The pooled findings should be interpreted with caution when extrapolated specifically to hypertensive populations with diabetes. Fourth, antihypertensive medication exposure was assessed at discrete study visits and less frequently than BP measurements; thus, interim regimen changes may have been misclassified. Moreover, SPRINT employed both attended and unattended automated office BP measurements, whereas ACCORD-BP used only attended measurements, though VIM-based BPV estimation mitigates this influence, a residual contribution to between-trial heterogeneity cannot be excluded. Finally, the relatively short duration of follow-up, low event rates, and the high-risk nature of the trial populations may have limited statistical power for detecting differences in clinical outcomes and may restrict generalizability to broader hypertensive populations. Given that this posthoc analysis involved further partitioning into matched subgroups across multiple pairwise regimen comparisons, the effective statistical power for each individual comparison was further reduced.

### Conclusions

Among high-risk hypertensive patients managed according to contemporary BP treatment paradigms derived from randomized BP target trials, CCB-based regimens were associated with lower visit-to-visit systolic BPV compared with both RAS inhibitor-based and diuretic-based regimens, despite similar mean BP control. However, these differences in BPV did not translate into corresponding differences in MACE. Further investigations are warranted to define personalized antihypertensive strategies that optimize both mean BP and variability while minimizing long-term cardiovascular risk.

## Article Information

### Acknowledgments

The authors would like to thank the SPRINT (Systolic Blood Pressure Intervention Trial) and ACCORD (Action to Control Cardiovascular Risk in Diabetes study participants and trial investigators. This article was prepared using data from SPRINT and ACCORD trials, obtained from the National Heart, Lung, and Blood Institute (NHLBI) Biologic Specimen and Data Repository Information Coordinating Centre. The views expressed are those of the authors and do not necessarily represent the official position of the NHLBI, the National Institutes of Health, or the ACCORD trial and SPRINT investigation groups.

### Disclosures

None.

### Supplemental Material

Tables S1–S7

Figures S1–S3

## Supplementary Material



## References

[1] NCD Risk Factor Collaboration (NCD-RisC). Worldwide trends in hypertension prevalence and progress in treatment and control from 1990 to 2019: a pooled analysis of 1201 population-representative studies with 104 million participants. Lancet. 2021;398:957–980. doi: 10.1016/S0140-6736(21)01330-1 34450083 10.1016/S0140-6736(21)01330-1 PMC8446938

[2] Ding R Elmadhoun O Chen R Ji X. Comprehensive strategies for stroke prevention: a narrative review of population-level risk factor mitigation [Published online October 9, 2025]. Brain Circ. 2025. doi: 10.4103/bc.bc_15_25 10.4103/bc.bc_15_25 PMC13524411 42667068

[3] Williams B Mancia G Spiering W Agabiti Rosei E Azizi M Burnier M Clement DL Coca A de Simone G Dominiczak A; ESC Scientific Document Group . 2018 ESC/ESH guidelines for the management of arterial hypertension. Eur Heart J. 2018;39:3021–3104. doi: 10.1093/eurheartj/ehy339 30165516 10.1093/eurheartj/ehy339

[4] Umemura S Arima H Arima S Asayama K Dohi Y Hirooka Y Horio T Hoshide S Ikeda S Ishimitsu T. The Japanese Society of Hypertension guidelines for the management of hypertension (JSH 2019). Hypertens Res. 2019;42:1235–1481. doi: 10.1038/s41440-019-0284-9 31375757 10.1038/s41440-019-0284-9

[5] Lukitasari M Jonnagaddala J Liaw ST Jalaludin B. Visit-to-visit blood pressure variability and cardiovascular outcomes: a systematic review and dose-response meta-analysis. Eur J Prev Cardiol. 2025;33:1127–1137. doi: 10.1093/eurjpc/zwaf546 10.1093/eurjpc/zwaf546 40874503

[6] Kulkarni S Parati G Bangalore S Bilo G Kim BJ Kario K Messerli F Stergiou G Wang J Whiteley W. Blood pressure variability: a review. J Hypertens. 2025;43:929–938. doi: 10.1097/HJH.0000000000003994 40084481 10.1097/HJH.0000000000003994 PMC12052075

[7] Unger T Borghi C Charchar F Khan NA Poulter NR Prabhakaran D Ramirez A Schlaich M Stergiou GS Tomaszewski M. 2020 International Society of Hypertension Global Hypertension Practice Guidelines. Hypertension. 2020;75:1334–1357. doi: 10.1161/HYPERTENSIONAHA.120.15026 32370572 10.1161/HYPERTENSIONAHA.120.15026

[8] Chen R Suchard MA Krumholz HM Schuemie MJ Shea S Duke J Pratt N Reich CG Madigan D You SC. Comparative First-line effectiveness and safety of ACE (Angiotensin-Converting Enzyme) inhibitors and angiotensin receptor blockers: a multinational cohort study. Hypertension. 2021;78:591–603. doi: 10.1161/HYPERTENSIONAHA.120.16667 34304580 10.1161/HYPERTENSIONAHA.120.16667 PMC8363588

[9] Abuhasira R Burrack N Turjeman A Patt YS Leibovici L Grossman A. Comparative analysis of first-line antihypertensive treatment classes. Am J Med. 2025;138:449–457.e7. doi: 10.1016/j.amjmed.2024.10.016 39424213 10.1016/j.amjmed.2024.10.016

[10] Parati G Bilo G Kollias A Pengo M Ochoa JE Castiglioni P Stergiou GS Mancia G Asayama K Asmar R. Blood pressure variability: methodological aspects, clinical relevance and practical indications for management - a European Society of Hypertension position paper. J Hypertens. 2023;41:527–544. doi: 10.1097/HJH.0000000000003363 36723481 10.1097/HJH.0000000000003363

[11] Buse JB Bigger JT Byington RP Cooper LS Cushman WC Friedewald WT Genuth S Gerstein HC Ginsberg HN Goff DC; ACCORD Study Group. Action to Control Cardiovascular Risk in Diabetes (ACCORD) trial: design and methods. Am J Cardiol. 2007;99:21i–33i. doi: 10.1016/j.amjcard.2007.03.003 10.1016/j.amjcard.2007.03.003 17599422

[12] Ambrosius WT Sink KM Foy CG Berlowitz DR Cheung AK Cushman WC Fine LJ Goff DC Johnson KC Killeen AA; SPRINT Study Research Group. The design and rationale of a multicenter clinical trial comparing two strategies for control of systolic blood pressure: the Systolic Blood Pressure Intervention Trial (SPRINT). Clin Trials. 2014;11:532–546. doi: 10.1177/1740774514537404 24902920 10.1177/1740774514537404 PMC4156910

[13] Whelton PK Carey RM Aronow WS Casey DE Collins KJ Dennison Himmelfarb C DePalma SM Gidding S Jamerson KA Jones DW. 2017 ACC/AHA/AAPA/ABC/ACPM/AGS/APhA/ASH/ASPC/NMA/PCNA guideline for the prevention, detection, evaluation, and management of high blood pressure in adults: executive summary: a report of the American College of Cardiology/American Heart Association Task Force on Clinical Practice Guidelines. Hypertension. 2018;71:1269–1324. doi: 10.1161/HYP.0000000000000066 29133354 10.1161/HYP.0000000000000066

[14] Wright JT Jr Williamson JD Whelton PK Snyder JK Sink KM Rocco MV Reboussin DM Rahman M Oparil S Lewis CE; SPRINT Research Group. A randomized trial of intensive versus standard blood-pressure control. N Engl J Med. 2015;373:2103–2116. doi: 10.1056/NEJMoa1511939 26551272 10.1056/NEJMoa1511939 PMC4689591

[15] Rothwell PM Howard SC Dolan E O’Brien E Dobson JE Dahlöf B Sever PS Poulter NR. Prognostic significance of visit-to-visit variability, maximum systolic blood pressure, and episodic hypertension. Lancet. 2010;375:895–905. doi: 10.1016/S0140-6736(10)60308-X 20226988 10.1016/S0140-6736(10)60308-X

[16] Group ACCRDS. Effects of intensive glucose lowering in type 2 diabetes. N Engl J Med. 2008;358:2545–2559. doi: 10.1056/NEJMoa0802743 18539917 10.1056/NEJMoa0802743 PMC4551392

[17] Wang JG Yan P Jeffers BW. Effects of amlodipine and other classes of antihypertensive drugs on long-term blood pressure variability: evidence from randomized controlled trials. J Am Soc Hypertens. 2014;8:340–349. doi: 10.1016/j.jash.2014.02.004 24685006 10.1016/j.jash.2014.02.004

[18] Kollias A Stergiou GS Kyriakoulis KG Bilo G Parati G. Treating visit-to-visit blood pressure variability to improve prognosis: Is amlodipine the drug of choice? Hypertension. 2017;70:862–866. doi: 10.1161/HYPERTENSIONAHA.117.10087 28947617 10.1161/HYPERTENSIONAHA.117.10087

[19] Gregg LP Hedayati SS Yang H Van Buren PN Banerjee S Navaneethan SD Virani SS Winkelmayer WC Alvarez CA. Association of blood pressure variability and diuretics with cardiovascular events in patients with chronic kidney disease stages 1-5. Hypertension. 2021;77:948–959. doi: 10.1161/HYPERTENSIONAHA.120.16117 33423525 10.1161/HYPERTENSIONAHA.120.16117 PMC7878423

[20] Muntner P Whittle J Lynch AI Colantonio LD Simpson LM Einhorn PT Levitan EB Whelton PK Cushman WC Louis GT. Visit-to-visit variability of blood pressure and coronary heart disease, stroke, heart failure, and mortality: a cohort study. Ann Intern Med. 2015;163:329–338. doi: 10.7326/M14-2803 26215765 10.7326/M14-2803 PMC5021508

[21] Spallone V. Blood pressure variability and autonomic dysfunction. Curr Diab Rep. 2018;18:137. doi: 10.1007/s11892-018-1108-z 30361834 10.1007/s11892-018-1108-z

[22] Chang TI Reboussin DM Chertow GM Cheung AK Cushman WC Kostis WJ Parati G Raj D Riessen E Shapiro B; SPRINT Research Group. Visit-to-visit office blood pressure variability and cardiovascular outcomes in SPRINT (Systolic Blood Pressure Intervention Trial). Hypertension. 2017;70:751–758. doi: 10.1161/HYPERTENSIONAHA.117.09788 28760939 10.1161/HYPERTENSIONAHA.117.09788 PMC6209591

[23] Cheng Y Li J Ren X Wang D Yang Y Miao Y Sheng C-S Tian J. Visit-to-visit office blood pressure variability combined with Framingham risk score to predict all-cause mortality: a post hoc analysis of the systolic blood pressure intervention trial. J Clin Hypertens (Greenwich). 2021;23:1516–1525. doi: 10.1111/jch.14314 34216524 10.1111/jch.14314 PMC8678842

[24] Mezue K Goyal A Pressman GS Matthew R Horrow JC Rangaswami J. Blood pressure variability predicts adverse events and cardiovascular outcomes in SPRINT. J Clin Hypertens (Greenwich). 2018;20:1247–1252. doi: 10.1111/jch.13346 29984884 10.1111/jch.13346 PMC8031192

[25] Rothwell PM. Limitations of the usual blood-pressure hypothesis and importance of variability, instability, and episodic hypertension. Lancet. 2010;375:938–948. doi: 10.1016/S0140-6736(10)60309-1 20226991 10.1016/S0140-6736(10)60309-1

[26] Schillaci G Pucci G Parati G. Blood pressure variability: an additional target for antihypertensive treatment? Hypertension. 2011;58:133–135. doi: 10.1161/HYPERTENSIONAHA.111.175752 21747045 10.1161/HYPERTENSIONAHA.111.175752

[27] Georgakis MK Gill D Webb AJS Evangelou E Elliott P Sudlow CLM Dehghan A Malik R Tzoulaki I Dichgans M. Genetically determined blood pressure, antihypertensive drug classes, and risk of stroke subtypes. Neurology. 2020;95:e353–e361. doi: 10.1212/WNL.0000000000009814 32611631 10.1212/WNL.0000000000009814 PMC7455321

[28] Rothwell PM Howard SC Dolan E O’Brien E Dobson JE Dahlöf B Poulter NR Sever PS; ASCOT-BPLA and MRC Trial Investigators. Effects of beta blockers and calcium-channel blockers on within-individual variability in blood pressure and risk of stroke. Lancet Neurol. 2010;9:469–480. doi: 10.1016/S1474-4422(10)70066-1 20227347 10.1016/S1474-4422(10)70066-1

[29] Yu J Song Q Bai J Wu S Bu P Li YF Cai J. Visit-to-visit blood pressure variability and cardiovascular outcomes in patients receiving intensive versus standard blood pressure control: insights from the STEP trial. Hypertension. 2023;80:1507–1516. doi: 10.1161/HYPERTENSIONAHA.122.20376 37170806 10.1161/HYPERTENSIONAHA.122.20376

[30] Gupta A Whiteley WN Godec T Rostamian S Ariti C Mackay J Whitehouse A Janani L Poulter NR Sever PS; ASCOT-10 Investigators . Legacy benefits of blood pressure treatment on cardiovascular events are primarily mediated by improved blood pressure variability: the ASCOT trial. Eur Heart J. 2024;45:1159–1169. doi: 10.1093/eurheartj/ehad814 38291599 10.1093/eurheartj/ehad814 PMC10984564

[31] Basson MD Newman WE Klug MG. Correlations among visit-to-visit blood pressure variability and treatment with antihypertensive medication with long-term adverse outcomes in a large veteran cohort. Am J Hypertens. 2021;34:1092–1099. doi: 10.1093/ajh/hpab087 34115112 10.1093/ajh/hpab087 PMC8557467

[32] Hara A Thijs L Asayama K Jacobs L Wang JG Staessen JA. Randomised double-blind comparison of placebo and active drugs for effects on risks associated with blood pressure variability in the Systolic Hypertension in Europe trial. PLoS One. 2014;9:e103169. doi: 10.1371/journal.pone.0103169 25090617 10.1371/journal.pone.0103169 PMC4121168

[33] Hoshide S Yano Y Mizuno H Kanegae H Kario K. Day-by-day variability of home blood pressure and incident cardiovascular disease in clinical practice: the J-HOP study (Japan Morning Surge-Home Blood Pressure). Hypertension. 2018;71:177–184. doi: 10.1161/HYPERTENSIONAHA.117.10385 29133364 10.1161/HYPERTENSIONAHA.117.10385

