# Supplementary material for: Blood Pressure Variability and Outcomes Across Antihypertensive Regimens
Source: Hypertension. 2026 Jun 25;83(9):e27140. doi: 10.1161/HYPERTENSIONAHA.126.27140 (PMC13480333; doi:10.1161/HYPERTENSIONAHA.126.27140)
Supplement: Supplementary file 1 [file hyp-83-e27140-s001.docx]

**Title Page**

**Blood Pressure Variability and Outcomes Across Antihypertensive Regimens**

**Short title:** BPV and Outcomes by Antihypertensive Regimen

Yue Qiao M.D.^1#^, Zihan Sun MSc^2#^, Eric L Harshfield Ph.D.^2^, Xunming Ji M.D., Ph.D.^3^, Hugh S Markus DM^2*^, Wenbo Zhao M.D., Ph.D.^1,2*^

1 Department of Neurology, Xuanwu Hospital, Capital Medical University, Beijing, China.

2 Stroke Research Group, Department of Clinical Neurosciences, University of Cambridge, Cambridge, United Kingdom.

^3^ Beijing Institute for Brain Disorders, Capital Medical University, Beijing, China.

^#^These authors contributed equally to this work as co-first authors.

^*^These authors contributed equally to this work as co-senior and co-corresponding authors.

***Correspondence to:**

Wenbo Zhao, MD, PhD, Department of Neurology, Xuanwu Hospital, Capital Medical University, No. 45, Changchun Street, Xicheng District, Beijing, 100053, China. Tel: +86-10-8319 9086. Fax: +86-10-83154745. Email: [zhaowb@xwh.ccmu.edu.cn](mailto:zhaowb@xwh.ccmu.edu.cn).

Hugh S. Markus, DM, Stroke Research Group, Department of Clinical Neurosciences, University of Cambridge, R3 Box 83, Cambridge Biomedical Campus, Cambridge, CB2 0QQ, UK. Tel: 01223 586661. Fax: 01223 217909. E-mail: [hsm32@medschl.cam.ac.uk](mailto:hsm32@medschl.cam.ac.uk).

**Supplemental materials**

**Supplementary Table S1. The baseline characteristics for person-trials in the emulated target trial after propensity score matching comparing monotherapy therapy regimens.**

| **Variable** | **RAS vs. Diuretic** | | | **RAS vs. CCB** | | | **Diuretic vs. CCB** | | | |
| --- | --- | --- | --- | --- | --- | --- | --- | --- | --- | --- |
|  | **RAS (n=482)** | **Diuretic (n=482)** | **SMD** | **RAS (n=347)** | **Diuretic (n=347)** | **SMD** | **Diuretic (n=336)** | **CCB (n=336)** | **SMD** |  |
| Intensive blood pressure control, No. (%) | 157 (32.5) | 144 (29.8) | 0.058 | 96 (27.7) | 96 (27.7) | <0.001 | 101 (30.1) | 96 (28.6) | 0.033 |  |
| Age, median (IQR) | 65.0 (59.0 -73.5) | 65.0 (59.0-74.0) | 0.035 | 67.0 (61.0-76.0) | 66.0 (60.0-77.0) | 0.012 | 66.0 (60.0-75.0) | 65.0 (60.0-76.0) | 0.017 |  |
| Female sex, No. (%) | 184 (38.1) | 198 (41.0) | 0.059 | 120 (34.6) | 114 (32.9) | 0.037 | 112 (33.3) | 113 (33.6) | 0.006 |  |
| BMI, median (IQR) | 29.8 (26.3- 33.9) | 29.8 (26.0-34.3) | 0.015 | 28.5 (25.5-31.9) | 28.0 (25.2-31.5) | 0.039 | 28.5 (25.3-31.8) | 28.4 (25.4-31.7) | 0.068 |  |
| Race/ethnicity, No. (%) |  |  | 0.022 |  |  | 0.063 |  |  | 0.049 |  |
| White | 288 (59.6) | 287 (59.4) |  | 180 (51.9) | 182 (52.4) |  | 173 (51.5) | 168 (50.0) |  |  |
| Black | 146 (30.2) | 146 (30.2) |  | 125 (36.0) | 120 (34.6) |  | 133 (39.6) | 134 (39.9) |  |  |
| Hispanic | 29 (6.0) | 28 (5.8) |  | 30 (8.6) | 35 (10.1) |  | 22 (6.5) | 26 (7.7) |  |  |
| Other | 20 (4.1) | 22 (4.6) |  | 12 (3.5) | 10 (2.9) |  | 8 (2.4) | 8 (2.4) |  |  |
| Education, No. (%) |  |  | 0.124 |  |  | 0.130 |  |  | 0.159 |  |
| Less than high school graduate | 38 (7.9) | 38 (7.9) |  | 22 (6.3) | 25 (7.2) |  | 29 (8.6) | 22 (6.5) |  |  |
| High school graduate or GED | 97 (20.1) | 78 (16.1) |  | 68 (19.6) | 63 (18.2) |  | 60 (17.9) | 58 (17.3) |  |  |
| Some college/technical school | 153 (31.7) | 176 (36.4) |  | 112 (32.3) | 131 (37.8) |  | 109 (32.4) | 133 (39.6) |  |  |
| College graduate or more | 195 (40.4) | 191 (39.5) |  | 145 (41.8) | 128 (36.9) |  | 138 (41.1) | 123 (36.6) |  |  |
| Diabetes, No. (%)* | 127 (26.3) | 120 (24.8) | 0.033 | 28 (8.0) | 25 (7.2) | 0.033 | 21 (6.2) | 20 (6.0) | 0.012 |  |
| Heart failure, No. (%) | 10 (2.1) | 9 (1.9) | 0.015 | 4 (1.2) | 5 (1.4) | 0.025 | 5 (1.5) | 5 (1.5) | <0.001 |  |
| History of cardiovascular disease, No. (%) | 65 (13.5) | 59 (12.2) | 0.037 | 40 (11.5) | 44 (12.7) | 0.035 | 36 (10.7) | 36 (10.7) | <0.001 |  |
| History of stroke, No. (%) | 4 (0.8) | 7 (1.4) | 0.059 | 2 (0.6) | 1 (0.3) | 0.044 | 1 ( 0.3) | 1 ( 0.3) | <0.001 |  |
| Current smoking, No. (%) | 58 (12.0) | 57 (11.8) | 0.006 | 44 (12.7) | 49 (14.1) | 0.042 | 56 (16.7) | 52 (15.5) | 0.032 |  |
| Baseline systolic blood pressure, mean (SD) | 136.5 (13.9) | 136.7 (14.0) | 0.012 | 136.0 (13.7) | 136.5 (12.5) | 0.043 | 136.5 (14.0) | 136.1 (12.8) | 0.027 |  |
| Baseline diastolic blood pressure, mean (SD) | 77.5 (10.5) | 77.8 (11.1) | 0.028 | 77.4 (11.1) | 77.7 (10.5) | 0.03 | 77.9 (11.2) | 78.0 (10.5) | 0.002 |  |
| eGFR, median (IQR) | 75.5 (64.9-90.1) | 76.5 (64.1-90.3) | 0.024 | 73.6 (61.9-90.0) | 74.9 (60.9-88.5) | 0.04 | 75.2 (63.1-86.5) | 74.1 (61.0-87.2) | 0.023 |  |
| Baseline serum creatine, median (IQR) | 1.0 (0.8-1.1) | 1.0 (0.8-1.1) | <0.001 | 1.0 (0.8-1.2) | 1.0 (0.8-1.2) | 0.048 | 1.0 (0.9-1.16) | 1.0 (0.9-1.2) | 0.029 |  |
| Baseline total cholesterol, median (IQR) | 196.0 (169.5-221.0) | 194.0 (169.5-221.0) | 0.013 | 192.0 (166.5-221.5) | 195.0 (168.5-221.0) | 0.006 | 97.0 (90.0-106.0) | 97.0 (90.0-106.0) | 0.060 |  |
| HDL cholesterol, median (IQR) | 50.0 (42.5-61.0) | 50.0 (43.0-60.0) | 0.015 | 52.0 (44.0-62.0) | 52.0 (43.0-63.0) | 0.003 | 52.0 (44.0-63.0-0) | 52.0 (44.0-64.0) | 0.005 |  |
| Glucose, median (IQR) | 101.0 (92.0-128.0) | 101.0 (93.0-120.5) | 0.043 | 96.0 (90.5-105.0) | 97.0 (89.0-106.0) | 0.011 | 194.5 (171.0-220.0) | 195.5 (170.0-222.0) | 0.007 |  |
| Baseline albumin/creatinine ratio, median (IQR) | 7.8 (5.0-16.5) | 8.6 (5.0-16.3) | 0.027 | 7.9 (5.0-15.9) | 8.5 (5.6-18.7) | 0.053 | 8.0 (5.0-14.5) | 8.2 (5.3-15.0) | 0.047 |  |
| Statin use, No. (%) | 182 (37.7) | 191 (39.5) | 0.038 | 140 (40.3) | 131 (37.8) | 0.053 | 120 (35.7) | 128 (38.1) | 0.049 |  |
| Aspirin use, No. (%) | 212 (43.9) | 226 (46.8) | 0.058 | 154 (44.4) | 140 (40.3) | 0.082 | 137 (40.8) | 140 (41.7) | 0.018 |  |

***Abbreviations:*** SMD, standardized mean difference; RAS, renin-angiotensin system inhibitors (angiotensin-converting enzyme inhibitors and angiotensin receptor blockers); CCB, calcium channel blockers; ITT, intention-to-treat; BMI, body mass index; eGFR, estimated glomerular filtration rate; HDL, high-density lipoprotein; GED, general educational development; SD, standard deviation; IQR, interquartile range.

*Diabetes refers to type 2 diabetes mellitus in the ACCORD-BP trial.

**Supplementary Table S2. The baseline characteristics for person-trials in the emulated target trial after propensity score matching comparing dual therapy regimens.**

| **Variable** | **RAS+Diuretic vs. RAS+CCB** | | | **RAS+Diuretic vs CCB+Diuretic** | | | **RAS+CCB vs CCB+Diuretic** | | |
| --- | --- | --- | --- | --- | --- | --- | --- | --- | --- |
|  | **RAS+Diuretic (n=746)** | **RAS+CCB (n=746)** | **SMD** | **RAS+Diuretic (n=456)** | **CCB+Diuretic (n=456)** | **SMD** | **RAS+CCB (n=387)** | **CCB+Diuretic (n=387)** | **SMD** |
| Intensive blood pressure control, No. (%) | 372 (49.9) | 362 (48.5) | 0.027 | 261 (57.2) | 239 (52.4) | 0.097 | 199 (51.4) | 196 (50.6) | 0.016 |
| Age, median (IQR) | 66.0 (60.0-75.0) | 65.0 (60.0-74.0) | 0.062 | 65.0 (60.0-73.0) | 66.0 (59.0-74.0) | 0.056 | 66.0 (60.0-75.0) | 66.0 (59.0-74.0) | 0.028 |
| Female sex, No. (%) | 288 (38.6) | 284 (38.1) | 0.011 | 183 (40.1) | 179 (39.3) | 0.018 | 153 (39.5) | 151 (39.0) | 0.011 |
| BMI, median (IQR) | 28.71 (25.8-32.4) | 28.4 (25.6-32.1) | 0.005 | 29.5 (26.6-33.5) | 29.6 (26.6-33.7) | 0.014 | 29.0 (25.9-32.5) | 29.0 (26.3-32.9) | 0.005 |
| Race/ethnicity, No. (%) |  |  | 0.017 |  |  | 0.026 |  |  | 0.106 |
| White | 411 (55.1) | 405 (54.3) |  | 222 (48.7) | 222 (48.7) |  | 166 (42.9) | 173 (44.7) |  |
| Black | 217 (29.1) | 220 (29.5) |  | 37 (8.1) | 35 (7.7) |  | 29 ( 7.5) | 34 (8.8) |  |
| Hispanic | 93 (12.5) | 95 (12.7) |  | 5 (1.1) | 6 (1.3) |  | 3 ( 0.8) | 6 (1.6) |  |
| Other | 25 (3.4) | 26 (3.5) |  | 192 (42.1) | 193 (42.3) |  | 189 (48.8) | 174 (45.0) |  |
| Education, No. (%) |  |  | 0.033 |  |  | 0.029 |  |  | 0.137 |
| Less than high school graduate | 64 (8.6) | 64 (8.6) |  | 50 (11.0) | 50 (11.0) |  | 35 (9.0) | 41 (10.6) |  |
| High school graduate or GED | 124 (16.6) | 125 (16.8) |  | 86 (18.9) | 87 (19.1) |  | 79 (20.4) | 62 (16.0) |  |
| Some college/technical school | 264 (35.4) | 253 (33.9) |  | 177 (38.8) | 171 (37.5) |  | 154 (39.8) | 149 (38.5) |  |
| College graduate or more | 294 (39.4) | 304 (40.8) |  | 143 (31.4) | 148 (32.5) |  | 119 (30.7) | 135 (34.9) |  |
| Diabetes, No. (%)* | 131 (17.5) | 129 (17.2) | 0.007 | 42 (9.2) | 42 (9.2) | <0.001 | 9 (2.3) | 6 (1.6) | 0.018 |
| Heart failure, No. (%) | 24 (3.2) | 18 (2.4) | 0.049 | 7 (1.5) | 8 (1.8) | 0.017 | 8 (2.1) | 7 (1.8) | 0.019 |
| History of cardiovascular disease, No. (%) | 125 (16.8) | 118 (15.8) | 0.025 | 46 (10.1) | 47 (10.3) | 0.007 | 51 (13.2) | 44 (11.4) | 0.055 |
| History of stroke, No. (%) | 8 (1.1) | 12 (1.6) | 0.047 | 7 (1.5) | 5 (1.1) | 0.038 | 2 (0.5) | 2 (0.5) | <0.001 |
| Current smoking, No. (%) | 85 (11.4) | 100 (13.4) | 0.061 | 72 (15.8) | 70 (15.4) | 0.012 | 62 (16.0) | 60 (15.5) | 0.014 |
| Baseline systolic blood pressure, mean (SD) | 139.4 (15.3) | 139.5 (14.8) | 0.004 | 140.9 (15.7) | 141.4 (14.7) | 0.035 | 140.3 (15.3) | 140.5 (14.5) | 0.013 |
| Baseline diastolic blood pressure, mean (SD) | 77.5 (11.1) | 78.0 (11.7) | 0.042 | 80.2 (11.4) | 80.4 (12.7) | 0.017 | 79.2 (12.0) | 79.4 (12.4) | 0.017 |
| eGFR, median (IQR) | 74.1 (60.6-88.8) | 73.3 (60.7-88.9) | 0.041 | 75.3 (61.8-87.6) | 74.7 (63.2-86.4) | 0.015 | 72.5 (60.7-87.0) | 74.8 (63.1-86.4) | 0.021 |
| Baseline serum creatine, median (IQR) | 1.0 (0.8, 1.2) | 1.0 (0.8-1.2) | 0.006 | 1.0 (0.9-1.2) | 1.0 (0.9-1.2) | 0.009 | 1.0 (0.9-1.2) | 1.0 (0.9-1.2) | 0.019 |
| Baseline total cholesterol, median (IQR) | 187.0 (164.0-215.8) | 187.0 (162.3- 214.0) | 0.024 | 192.0 (169.8-215.3) | 190.0 (166.0-219.0) | 0.010 | 189.0 (168.0, 217.5) | 191.0 (168.0, 219.0) | 0.002 |
| HDL cholesterol, median (IQR) | 51.0 (44.0-61.0) | 51.0 (43.0-62.0) | 0.003 | 52.0 (43.0-64.0) | 53.0 (44.0-63.0) | 0.013 | 52.0 (44.0-63.5) | 53.0 (44.0-63.5) | 0.006 |
| Glucose, median (IQR) | 98.0 (90.0-108.0) | 98.0 (91.0-109.0) | 0.027 | 97.0 (90.0-107.0) | 97.0 (91.0-107.3) | 0.016 | 98.0 (90.5-107.0) | 96.0 (90.5-106.0) | 0.008 |
| Baseline albumin/creatinine ratio, median (IQR) | 8.8 (5.4-19.1) | 10.2 (6.1-23.3) | 0.007 | 7.9 (5.0-15.3) | 9.9 (5.8-19.9) | 0.037 | 9.3 (6.0-18.0) | 10.2 (6.0-19.8) | 0.01 |
| Statin use, No. (%) | 343 (46.0) | 318 (42.6) | 0.068 | 147 (32.2) | 150 (32.9) | 0.014 | 134 (34.6) | 130 (33.6) | 0.022 |
| Aspirin use, No. (%) | 373 (50.0) | 350 (46.9) | 0.062 | 191 (41.9) | 193 (42.3) | 0.009 | 163 (42.1) | 168 (43.4) | 0.026 |

***Abbreviations:*** SMD, standardized mean difference; RAS, renin-angiotensin system inhibitors (angiotensin-converting enzyme inhibitors and angiotensin receptor blockers); CCB, calcium channel blockers; ITT, intention-to-treat; BMI, body mass index; eGFR, estimated glomerular filtration rate; HDL, high-density lipoprotein; GED, general educational development; SD, standard deviation; IQR, interquartile range.

*Diabetes refers to type 2 diabetes mellitus in the ACCORD-BP trial.

**Supplementary Table S3. Baseline Characteristics of the Unmatched Study Cohort**

| **Variable** | **RAS**  **N=1538** | **CCB**  **N=376** | **Diuretic**  **N=536** | **RAS+CCB**  **N=776** | **RAS+Diuretic**  **N=2092** | **CCB+Diuretic**  **N=461** |
| --- | --- | --- | --- | --- | --- | --- |
| Intensive blood pressure control, No. (%) | 444 (28.9) | 104 (27.7) | 159 (29.7) | 365 (47.0) | 1196 (57.2) | 242 (52.5) |
| Age, y, mean (SD) | 65.4 (8.7) | 68.0 (8.9) | 66.3 (9.3) | 66.9 (9.2) | 65.2 (8.4) | 66.88 (9.2) |
| Female sex, No. (%) | 553 (36.0) | 126 (33.5) | 231 (43.1) | 293 (37.8) | 910 (43.5) | 182 (39.5) |
| BMI, kg/m2, median (IQR) | 29.1 (26.0-32.7) | 27.9 (25.2-31.5) | 30.0 (26.0-35.0) | 28.2 (25.5-32.0) | 30.5 (27.0-35.0) | 29.6 (26.6-33.7) |
| Ethnicity, No. (%) |  |  |  |  |  |  |
| White | 995 (64.7) | 182 (48.4) | 291 (54.3) | 412 (53.1) | 1244 (59.5) | 193 (41.9) |
| Black | 255 (16.6) | 147 (39.1) | 194 (36.2) | 230 (29.6) | 545 (26.1) | 225 (48.8) |
| Hispanic | 183 (11.9) | 35 (9.3) | 28 (5.2) | 105 (13.5) | 187 (8.9) | 37 (8.0) |
| Other | 105 (6.8) | 12 (3.2) | 23 (4.3) | 29 (3.7) | 116 (5.5) | 6 (1.3) |
| Education, No. (%) |  |  |  |  |  |  |
| Less than high school graduate | 142 (9.2) | 30 (8.0) | 44 (8.2) | 67 (8.6) | 201 (9.6) | 53 (11.5) |
| High school graduate or GED | 305 (19.8) | 71 (18.9) | 88 (16.4) | 127 (16.4) | 429 (20.5) | 88 (19.1) |
| Some college/technical school | 490 (31.9) | 144 (38.3) | 200 (37.3) | 263 (33.9) | 742 (35.5) | 172 (37.3) |
| College graduate or more | 601 (39.1) | 131 (34.8) | 204 (38.1) | 319 (41.1) | 720 (34.4) | 148 (32.1) |
| Comorbid conditions, No. (%) |  |  |  |  |  |  |
| Hypertension | 1281 (83.3) | 335 (89.1) | 464 (86.6) | 726 (93.6) | 1845 (88.2) | 420 (91.1) |
| Diabetes | 656 (42.7) | 26 (6.9) | 132 (24.6) | 132 (17.0) | 838 (40.1) | 43 (9.3) |
| Heart failure | 16 (1.0) | 6 (1.6) | 10 (1.9) | 18 (2.3) | 42 (2.0) | 8 (1.7) |
| Atrial fibrillation | 51 (3.5) | 23 (6.2) | 18 (3.5) | 47 (6.2) | 57 (2.8) | 21 (4.6) |
| History of cardiovascular disease | 250 (16.3) | 47 (12.5) | 62 (11.6) | 133 (17.1) | 304 (14.5) | 47 (10.2) |
| History of stroke | 36 (2.3) | 1 (0.3) | 8 (1.5) | 16 (2.1) | 41 (2.0) | 6 (1.3) |
| Albuminuria | 193 (13.7) | 21 (5.7) | 49 (9.6) | 51 (6.9) | 252 (13.2) | 20 (4.4) |
| Smoking status, No. (%) |  |  |  |  |  |  |
| Never smoked | 769 (50.0) | 166 (44.1) | 232 (43.4) | 374 (48.2) | 995 (47.6) | 202 (43.8) |
| Former smoker | 600 (39.0) | 148 (39.4) | 229 (42.8) | 295 (38.0) | 842 (40.2) | 188 (40.8) |
| Current smoker | 168 (10.9) | 62 (16.5) | 74 (13.8) | 107 (13.8) | 255 (12.2) | 71 (15.4) |
| Baseline systolic blood pressure, mm Hg, mean (SD) | 135.7 (13.6) | 136.1 (12.9) | 136.6 (13.8) | 139.4 (14.7) | 139.5 (15.3) | 141.3 (14.6) |
| Baseline diastolic blood pressure, mm Hg, mean (SD) | 76.6 (10.2) | 77.4 (10.5) | 78.1 (11.2) | 77.8 (11.8) | 78.5 (11.0) | 80.2 (12.8) |
| eGFR, mL/min/1.73 m2, median (IQR) | 79.9 (66.6-93.1) | 74.9 (61.3-88.3) | 77.2 (64.5-90.4) | 73.9 (60.6-89.3) | 78.7 (65.8-92.5) | 74.3 (63.1-86.4) |
| Baseline serum creatine, (mg/dL), mean (SD) | 1.0 (0.3) | 1.0 (0.3) | 1.0 (0.3) | 1.04 (0.3) | 1.0 (0.3) | 1.1 (0.3) |
| Baseline Total cholesterol (mg/dL), median (IQR) | 191 (164.0-218.0) | 195 (169.0-221.0) | 196 (171.0-222.0) | 187 (162.5-214.0) | 191 (166.0-218.0) | 190 (166.0-219.0) |
| Low-density lipoprotein (mg/dL), mean (SD) | 114.4 (35.3) | 115.8 (33.9) | 120.3 (34.5) | 112.6 (34.4) | 114.3 (34.2) | 115.9 (33.9) |
| HDL cholesterol, (mg/dL), mean (SD) | 50.3 (14.4) | 55.0 (16.3) | 53.1 (14.6) | 53.6 (14.8) | 51.1 (14.3) | 54.6 (14.1) |
| Glucose (mg/dL), median (IQR) | 106 (94.0-156.0) | 97 (89.8-106.0) | 101 (93.0-120.0) | 98 (91.0-109.0) | 106 (94.0-149.0) | 97 (91.0-107.0) |
| Baseline albumin/creatinine ratio, median (IQR) | 9.0 (5.4-21.0) | 8.5 (5.5-18.8) | 8.8 (5.0-16.2) | 10.4 (6.1-24.3) | 9.1 (5.4-20.6) | 10.0 (5.7-20.4) |
| Statin use, No. (%) | 744 (48.8) | 138 (36.9) | 203 (38.2) | 330 (42.7) | 985 (47.3) | 149 (32.5) |
| Aspirin use, No. (%) | 832 (54.2) | 226 (60.3) | 288 (53.9) | 411 (53.0) | 1063 (51.0) | 265 (57.9) |
| Follow-up characteristics |  |  |  |  |  |  |
| No. of BP measurements for BPV calculation, median (IQR) | 10.0 (9.0-13.0) | 11.0 (9.0-13.0) | 11.0 (9.0-13.0) | 11.0 (9.0-14.0) | 11.0 (9.0-15.0) | 11.0 (9.0-13.0) |
| Duration of follow up, days, median (IQR) | 1291 (1095-1460) | 1305 (1116-1476) | 1310 (1095-1466) | 1290 (1095-1460) | 1288 (1095-1460) | 1297 (1129-1499) |
| Mean SBP during follow-up, mm Hg, mean (SD) | 129.0 (10.0) | 131.0 (9.4) | 129.9 (9.9) | 129.0 (10.8) | 125.8 (11.2) | 128.5 (10.1) |
| Major adverse cardiovascular events, No. (%) | 49 (3.2) | 10 (2.7) | 21 (3.9) | 41 (5.3) | 76 (3.6) | 18 (3.9) |

SD, standard deviation; IQR, interquartile range; BMI, body mass index; GED, general educational development; eGFR, estimated glomerular filtration rate; HDL, high-density lipoprotein; RAS, renin-angiotensin system inhibitors (angiotensin-converting enzyme inhibitors and angiotensin receptor blockers); CCB, calcium channel blockers; BP, blood pressure.

**Supplementary Table S4. Mean Systolic Blood Pressure During Follow-up in Matched Cohorts**

| **Pairwise Comparisons** | **Intention-to-Treat Cohort**  **Mean SBP±SD (mmHg)** | | | | **Per-Protocol Cohort**  **Mean SBP±SD (mmHg)** | | | |
| --- | --- | --- | --- | --- | --- | --- | --- | --- |
|  | **Regimen 1** | **Regimen 2 (Ref)** | **P value†** | **SMD** | **Regimen 1** | **Regimen 2 (Ref)** | **P value†** | **SMD** |
| Diuretic vs RAS (Ref) | 129.96±9.82 | 128.83±9.98 | 0.078 | 0.114 | 129.79±10.10 | 128.96±11.04 | 0.318 | 0.079 |
| CCB vs RAS (Ref) | 130.75±9.31 | 130.49±9.27 | 0.709 | 0.028 | 131.01±9.81 | 130.82±9.50 | 0.837 | 0.019 |
| CCB vs Diuretic (Ref) | 130.78±9.46 | 130.57±9.84 | 0.787 | 0.021 | 131.11±9.72 | 130.58±10.11 | 0.573 | 0.054 |
| RAS+CCB vs RAS+Diuretic (Ref) | 128.81±10.88 | 127.45±10.94 | 0.016 | 0.125 | 128.84±11.29 | 125.33±11.23 | <0.001 | 0.312 |
| CCB+Diuretic vs RAS+Diuretic (Ref) | 128.43±10.11 | 127.09±10.84 | 0.054 | 0.128 | 128.00±10.24 | 125.01±11.35 | 0.001 | 0.276 |
| CCB+Diuretic vs RAS+CCB (Ref) | 128.52±10.12 | 128.56±10.94 | 0.956 | 0.004 | 127.77±9.98 | 128.49±10.86 | 0.460 | 0.069 |

SBP, systolic blood pressure; SD, standard deviation; RAS, renin-angiotensin system inhibitors (angiotensin-converting enzyme inhibitors and angiotensin receptor blockers); CCB, calcium channel blockers.

Mean SBP values represent the average of all visit-to-visit SBP measurements obtained during the follow-up period, defined from the baseline medication assessment (3 months in SPRINT; 12 months in ACCORD-BP) to the occurrence of a major adverse cardiovascular event or end of follow-up, whichever occurred first. Comparisons were performed after 1:1 propensity score matching. P values were calculated using independent t tests for pairwise comparisons of mean SBP between matched treatment groups. Overall, mean SBP levels during follow-up were comparable across antihypertensive regimens in both intention-to-treat and per-protocol analyses, with the exception of comparisons between RAS inhibitor plus CCB versus RAS inhibitor plus diuretic (intention-to-treat: P=0.016; per-protocol: P<0.001) and between CCB plus diuretic versus RAS inhibitor plus diuretic in the per-protocol analysis (P=0.001).

**Table S5. Covariate-Adjusted Associations of Antihypertensive Regimens with Visit-to-Visit Systolic Blood Pressure Variability**

| **Comparison** | **BP metrics** | **Intention-to-treat (ITT)** | | **Per-protocol (PP)** | |
| --- | --- | --- | --- | --- | --- |
|  |  | **Adjusted β (95% CI)** | **Adjusted P** | **Adjusted β (95% CI)** | **Adjusted P** |
| Diuretic vs RAS (Ref) | SBP-SD | -0.469 (-1.001, 0.062) | 0.083 | -1.239 (-2.019, -0.459) | 0.002 |
|  | SBP-CV | -0.388 (-0.786, 0.010) | 0.056 | -0.877 (-1.467, -0.288) | 0.004 |
|  | SBP-ARV | -0.554 (-1.192, 0.083) | 0.088 | -1.024 (-2.034, -0.014) | 0.047 |
|  | SBP-VIM | -0.51 (-1.016, -0.004) | 0.048 | -1.155 (-1.905, -0.405) | 0.003 |
|  | SBP-Mean | 0.39 (-0.459, 1.238) | 0.368 | -0.409 (-1.527, 0.710) | 0.473 |
| CCB vs RAS (Ref) | SBP-SD | -1.243 (-1.846, -0.64) | <0.001 | -1.393 (-2.236, -0.550) | 0.001 |
|  | SBP-CV | -0.968 (-1.421, -0.515) | <0.001 | -1.057 (-1.698, -0.416) | 0.001 |
|  | SBP-ARV | -1.587 (-2.360, -0.815) | <0.001 | -1.676 (-2.733, -0.620) | 0.002 |
|  | SBP-VIM | -1.228 (-1.805, -0.652) | <0.001 | -1.362 (-2.175, -0.550) | 0.001 |
|  | SBP-Mean | 0.31 (-0.695, 1.314) | 0.545 | -0.416 (-1.745, 0.912) | 0.538 |
| CCB vs Diuretic (Ref) | SBP-SD | -0.915 (-1.489, -0.34) | 0.002 | -0.581 (-1.384, 0.222) | 0.155 |
|  | SBP-CV | -0.7 (-1.128, -0.272) | 0.001 | -0.472 (-1.091, 0.147) | 0.135 |
|  | SBP-ARV | -0.735 (-1.434, -0.036) | 0.039 | -1.038 (-2.030, -0.046) | 0.04 |
|  | SBP-VIM | -0.89 (-1.433, -0.346) | 0.001 | -0.598 (-1.384, 0.188) | 0.135 |
|  | SBP-Mean | 0.065 (-0.972, 1.103) | 0.902 | 0.049 (-1.289, 1.387) | 0.943 |
| RAS+CCB vs RAS+Diuretic (Ref) | SBP-SD | -0.495 (-0.945, -0.046) | 0.031 | -0.794 (-1.391, -0.196) | 0.009 |
|  | SBP-CV | -0.42 (-0.751, -0.089) | 0.013 | -0.675 (-1.123, -0.228) | 0.003 |
|  | SBP-ARV | -0.607 (-1.162, -0.052) | 0.032 | -1.013 (-1.761, -0.265) | 0.008 |
|  | SBP-VIM | -0.562 (-0.982, -0.142) | 0.009 | -0.853 (-1.422, -0.284) | 0.003 |
|  | SBP-Mean | 1.027 (0.323, 1.730) | 0.004 | 1.620 (0.721, 2.520) | <0.001 |
| CCB+Diuretic vs RAS+Diuretic (Ref) | SBP-SD | -1.220 (-1.798, -0.642) | <0.001 | -2.220 (-3.108, -1.332) | <0.001 |
|  | SBP-CV | -0.963 (-1.391, -0.536) | <0.001 | -1.775 (-2.438, -1.111) | <0.001 |
|  | SBP-ARV | -1.271 (-1.956, -0.586) | <0.001 | -2.309 (-3.456, -1.163) | <0.001 |
|  | SBP-VIM | -1.237 (-1.779, -0.696) | <0.001 | -2.256 (-3.100, -1.412) | <0.001 |
|  | SBP-Mean | 0.524 (-0.352, 1.400) | 0.241 | 1.010 (-0.262, 2.282) | 0.119 |
| CCB+Diuretic vs RAS+CCB (Ref) | SBP-SD | -0.693 (-1.259, -0.128) | 0.016 | -0.790 (-1.591, 0.010) | 0.053 |
|  | SBP-CV | -0.542 (-0.965, -0.118) | 0.012 | -0.600 (-1.202, 0.002) | 0.051 |
|  | SBP-ARV | -0.378 (-1.057, 0.301) | 0.275 | -0.373 (-1.420, 0.675) | 0.485 |
|  | SBP-VIM | -0.693 (-1.233, -0.153) | 0.012 | -0.754 (-1.520, 0.012) | 0.054 |
|  | SBP-Mean | -0.118 (-1.100, 0.864) | 0.813 | -0.379 (-1.680, 0.923) | 0.568 |

BPV, blood pressure variability; RAS, renin-angiotensin system inhibitors (angiotensin-converting enzyme inhibitors and angiotensin receptor blockers); CCB, calcium channel blockers; SBP, systolic blood pressure; SD, standard deviation; CV, coefficient of variation; ARV, average real variability; VIM, variation independent of mean; CI, confidence interval; Ref, reference group; ITT, intention-to-treat; PP, per-protocol. Adjusted models additionally included age, sex, education, ethnicity, treatment allocation, baseline SBP, eGFR, history of cardiovascular disease, heart failure, statin use, and aspirin use.

**Table S6. Associations of Antihypertensive Regimens with Visit-to-Visit Systolic Blood Pressure Variability in the Intention-to-Treat Analysis (Separately by Study)**

| **Comparison** | **BP metrics** | **ACCORD-BP** | | **SPRINT** | | **Interaction P** |
| --- | --- | --- | --- | --- | --- | --- |
|  |  | **β (95% CI)** | **P** | **β (95% CI)** | **P** |  |
| Diuretic vs RAS (Ref) | SBP-SD | -1.149 (-2.404, 0.106) | 0.072 | -0.232 (-0.838, 0.373) | 0.451 | 0.162 |
|  | SBP-CV | -0.859 (-1.791, 0.073) | 0.071 | -0.227 (-0.677, 0.222) | 0.321 | 0.194 |
|  | SBP-ARV | -0.791 (-2.228, 0.646) | 0.279 | -0.452 (-1.188, 0.285) | 0.229 | 0.664 |
|  | SBP-VIM | -1.094 (-2.279, 0.092) | 0.070 | -0.311 (-0.883, 0.261) | 0.286 | 0.206 |
|  | SBP-Mean | 0.285 (-2.507, 3.076) | 0.841 | 0.474 (-0.910, 1.858) | 0.502 | 0.899 |
| CCB vs RAS (Ref) | SBP-SD | -0.457 (-2.989, 2.075) | 0.717 | -1.304 (-1.946, -0.661) | <0.001 | 0.524 |
|  | SBP-CV | -0.656 (-2.526, 1.214) | 0.482 | -1.006 (-1.485, -0.526) | <0.001 | 0.725 |
|  | SBP-ARV | 0.653 (-2.448, 3.754) | 0.673 | -1.715 (-2.543, -0.887) | <0.001 | 0.166 |
|  | SBP-VIM | -0.785 (-3.164, 1.595) | 0.509 | -1.283 (-1.893, -0.673) | <0.001 | 0.693 |
|  | SBP-Mean | 6.094 (-0.931, 13.120) | 0.087 | 0.120 (-1.303, 1.543) | 0.869 | 0.046 |
| CCB vs Diuretic (Ref) | SBP-SD | -1.172 (-3.765, 1.421) | 0.363 | -1.019 (-1.646, -0.393) | 0.001 | 0.916 |
|  | SBP-CV | -0.954 (-2.959, 1.051) | 0.339 | -0.778 (-1.239, -0.317) | <0.001 | 0.870 |
|  | SBP-ARV | -1.309 (-4.857, 2.239) | 0.457 | -0.944 (-1.699, -0.189) | 0.014 | 0.836 |
|  | SBP-VIM | -1.204 (-3.739, 1.330) | 0.340 | -0.991 (-1.574, -0.407) | <0.001 | 0.875 |
|  | SBP-Mean | 1.010 (-6.519, 8.538) | 0.786 | 0.106 (-1.361, 1.574) | 0.887 | 0.793 |
| RAS+CCB vs RAS+Diuretic (Ref) | SBP-SD | -0.867 (-2.119, 0.386) | 0.174 | -0.481 (-0.987, 0.026) | 0.063 | 0.555 |
|  | SBP-CV | -0.557 (-1.468, 0.355) | 0.230 | -0.436 (-0.806, -0.066) | 0.021 | 0.800 |
|  | SBP-ARV | -1.188 (-2.703, 0.327) | 0.124 | -0.565 (-1.196, 0.067) | 0.080 | 0.441 |
|  | SBP-VIM | -0.724 (-1.885, 0.438) | 0.221 | -0.585 (-1.054, -0.115) | 0.015 | 0.818 |
|  | SBP-Mean | -0.423 (-3.313, 2.466) | 0.773 | 1.296 (0.102, 2.490) | 0.033 | 0.262 |
| CCB+Diuretic vs RAS+Diuretic (Ref) | SBP-SD | -1.260 (-3.701, 1.180) | 0.306 | -1.140 (-1.757, -0.523) | <0.001 | 0.916 |
|  | SBP-CV | -1.084 (-2.833, 0.666) | 0.221 | -0.931 (-1.383, -0.479) | <0.001 | 0.856 |
|  | SBP-ARV | -0.997 (-4.585, 2.592) | 0.581 | -1.173 (-1.894, -0.453) | 0.001 | 0.898 |
|  | SBP-VIM | -1.359 (-3.594, 0.876) | 0.229 | -1.215 (-1.787, -0.642) | <0.001 | 0.892 |
|  | SBP-Mean | 1.699 (-3.343, 6.742) | 0.504 | 1.063 (-0.349, 2.475) | 0.140 | 0.807 |
| CCB+Diuretic vs RAS+CCB (Ref) | SBP-SD | 0.384 (-2.251, 3.019) | 0.772 | -0.808 (-1.409, -0.207) | 0.009 | 0.296 |
|  | SBP-CV | 0.188 (-1.682, 2.058) | 0.841 | -0.633 (-1.086, -0.180) | 0.006 | 0.336 |
|  | SBP-ARV | 2.207 (-1.619, 6.033) | 0.253 | -0.640 (-1.346, 0.067) | 0.076 | 0.039 |
|  | SBP-VIM | 0.258 (-2.130, 2.646) | 0.829 | -0.815 (-1.395, -0.234) | 0.006 | 0.326 |
|  | SBP-Mean | 1.206 (-5.389, 7.801) | 0.716 | -0.082 (-1.622, 1.459) | 0.917 | 0.658 |

BPV, blood pressure variability; RAS, renin-angiotensin system inhibitors (angiotensin-converting enzyme inhibitors and angiotensin receptor blockers); CCB, calcium channel blockers; SBP, systolic blood pressure; SD, standard deviation; CV, coefficient of variation; ARV, average real variability; VIM, variation independent of mean; CI, confidence interval; Ref, reference group; ITT, intention-to-treat.

**Table S7. Drug Class and MACE Outcomes — Cox Proportional Hazards Regression (ITT, Separately by Study)**

| **Comparison** | **Outcome** | **ACCORD-BP** | | | | **SPRINT** | |  |  | **Interaction P** |
| --- | --- | --- | --- | --- | --- | --- | --- | --- | --- | --- |
|  |  | **No. of participants per group** | **Events/ Rate per 100 person year** | **HR (95% CI)** | **P** | **No. of participants per group** | **Events/ Rate per 100 person year** | **HR(95% CI)** | **P** |  |
| Diuretic vs RAS (Ref) | MACE | 116 | 8(1.704) vs  6(1.259) | 1.376 (0.477–3.967) | 0.555 | 367 | 13(0.995) vs 10(0.757) | 1.321 (0.579–3.012) | 0.508 | 0.951 |
|  | Stroke | 116 | 4(0.843) vs 1(0.207) | 4.067 (0.455–36.388) | 0.210 | 367 | 4(0.303) vs 5(0.373) | 0.817 (0.219–3.041) | 0.763 | 0.216 |
|  | MI | 116 | 3(0.631) vs 3(0.626) | 1.017 (0.205–5.038) | 0.984 | 367 | 5(0.474) vs 6(0.564) | 0.849 (0.259–2.782) | 0.787 | 0.855 |
|  | CVD Death | 116 | 2(0.416) vs 3(0.617) | 0.702 (0.117–4.205) | 0.698 | 367 | - | - | 0.999 | - |
| CCB vs RAS (Ref) | MACE | 21 | 0(0) vs 3(3.477) | - | - | 327 | 9(0.78) vs 11(0.946) | 0.826 (0.342–1.993) | 0.670 | - |
|  | Stroke | 21 | 0(0) vs 0(0) | - | - | 327 | 3(0.257) vs 7(0.595) | 0.431 (0.111–1.667) | 0.223 | - |
|  | MI | 21 | 0(0) vs 3(3.474) | - | - | 327 | 3(0.326) vs 5(0.534) | 0.616 (0.147–2.579) | 0.507 | - |
|  | CVD Death | 21 | 0(0) vs 0(0) | - | - | 327 | - | - | 0.999 | - |
| CCB vs Diuretic (Ref) | MACE | 16 | 1(1.512) vs 0(0) | - | - | 320 | 9(0.789) vs 9(0.794) | 0.996 (0.395–2.509) | 0.993 | - |
|  | Stroke | 16 | 0(0) vs 0(0) | - | - | 320 | 2(0.174) vs 3(0.261) | 0.666 (0.111–3.987) | 0.656 | - |
|  | MI | 16 | 1(1.511) vs 0(0) | - | - | 320 | 3(0.328) vs 4(0.442) | 0.720 (0.161–3.218) | 0.667 | - |
|  | CVD Death | 16 | 0(0) vs 0(0) | - | - | 320 | 4(0.322) vs 3(0.242) | 1.336 (0.299–5.969) | 0.705 | - |
| RAS+CCB vs RAS+Diuretic (Ref) | MACE | 120 | 11(2.229) vs 3(0.617) | 3.393 (0.945–12.182) | 0.061 | 628 | 29(1.312) vs 23(1.032) | 1.264 (0.731–2.184) | 0.402 | 0.161 |
|  | Stroke | 120 | 3(0.599) vs 1(0.205) | 2.795 (0.290–26.942) | 0.374 | 628 | 11(0.488) vs 10(0.443) | 1.115 (0.473–2.627) | 0.803 | 0.458 |
|  | MI | 120 | 5(1.01) vs 1(0.205) | 4.602 (0.537–39.449) | 0.164 | 628 | 15(0.843) vs 9(0.501) | 1.662 (0.727–3.799) | 0.228 | 0.383 |
|  | CVD Death | 120 | 5(0.991) vs 1(0.204) | 4.442 (0.518–38.096) | 0.174 | 628 | 4(0.164) vs 6(0.246) | 0.660 (0.186–2.340) | 0.520 | 0.130 |
| CCB+Diuretic vs RAS+Diuretic (Ref) | MACE | 35 | 3(2.097) vs 1(0.652) | 3.167 (0.329–30.468) | 0.318 | 420 | 14(0.922) vs 14(0.923) | 0.995 (0.474–2.087) | 0.989 | 0.335 |
|  | Stroke | 35 | 1(0.691) vs 0(0) | - | - | 420 | 3(0.195) vs 6(0.391) | 0.502 (0.125–2.007) | 0.330 | - |
|  | MI | 35 | 2(1.379) vs 1(0.651) | 2.148 (0.195–23.715) | 0.533 | 420 | 9(0.747) vs 6(0.495) | 1.499 (0.534–4.213) | 0.442 | 0.791 |
|  | CVD Death | 35 | 0(0) vs 0(0) | - | - | 420 | 2(0.121) vs 3(0.182) | 0.655 (0.109–3.920) | 0.643 | - |
| CCB+Diuretic vs RAS+CCB (Ref) | MACE | 29 | 3(2.574) vs 2(1.614) | 1.616 (0.270–9.683) | 0.599 | 358 | 13(1.006) vs 16(1.26) | 0.801 (0.386–1.666) | 0.553 | 0.476 |
|  | Stroke | 29 | 1(0.846) vs 0(0) | - | - | 358 | 2(0.152) vs 3(0.23) | 0.645 (0.108–3.864) | 0.631 | - |
|  | MI | 29 | 2(1.687) vs 2(1.612) | 1.071 (0.151–7.612) | 0.945 | 358 | 9(0.881) vs 11(1.075) | 0.830 (0.344–2.002) | 0.678 | 0.812 |
|  | CVD Death | 29 | 0(0) vs 1(0.788) | - | - | 358 | 2(0.143) vs 2(0.142) | 1.001 (0.141–7.109) | 0.999 | - |

***Abbreviation:*** RAS, renin-angiotensin system inhibitors (angiotensin-converting enzyme inhibitors and angiotensin receptor blockers); CCB, calcium channel blockers; MACE, major adverse cardiovascular events; MI, myocardial infarction; CVD Death, cardiovascular death; HR, hazard ratio; CI, confidence interval; ITT, intention-to-treat.

**Supplementary Figure S1. Propensity Score Distribution After Matching.**

Density plots of propensity score distributions for each of the six pairwise antihypertensive regimen comparisons. A: Diuretic vs. RAS; B: CCB vs. RAS; C: CCB vs. Diuretic; D: RAS+CCB vs. RAS+Diuretic; E: CCB+Diuretic vs. RAS+Diuretic; F: CCB+Diuretic vs. RAS+CCB. In each panel, the overlapping shaded areas represent the propensity score distributions of the two treatment groups, with greater overlap indicating improved covariate balance following matching. This propensity score matching was used for both intention-to-treat and per-protocol analyses.

RAS, renin-angiotensin system inhibitors (angiotensin-converting enzyme inhibitors and angiotensin receptor blockers); CCB, calcium channel blockers.

**Supplementary Figure S2. Cumulative Incidence of Major Adverse Cardiovascular Events by Antihypertensive Regimen in the Intention-to-Treat Analysis**

Kaplan-Meier cumulative incidence curves for the composite outcome of major adverse cardiovascular events (MACE), defined as non-fatal myocardial infarction, non-fatal stroke, or cardiovascular death, across six antihypertensive regimen groups in the intention-to-treat cohort. RAS, renin-angiotensin system inhibitors (angiotensin-converting enzyme inhibitors and angiotensin receptor blockers); CCB, calcium channel blockers.

**Supplementary Figure S3. Antihypertensive Regimens and Major Adverse Cardiovascular Events in the Per-Protocol Analysis**

Forest plot of crude and covariate-adjusted hazard ratios (95% CI) for MACE and its individual components across six pairwise antihypertensive regimen comparisons in the propensity score-matched Per-Protocol cohort. Events are presented as n (rate per 100 person year). Adjusted models included age, sex, education, ethnicity, treatment allocation, baseline systolic blood pressure, eGFR, history of cardiovascular disease, heart failure, statin use, and aspirin use.

***Abbreviation:*** RAS, renin-angiotensin system inhibitors (angiotensin-converting enzyme inhibitors and angiotensin receptor blockers); CCB, calcium channel blockers; MACE, major adverse cardiovascular events; MI, myocardial infarction; CVD Death, cardiovascular death; HR, hazard ratio; CI, confidence interval.
